# Epidemiological evidence relating snus to health – an updated review based on recent publications

**DOI:** 10.1186/1477-7517-10-36

**Published:** 2013-12-06

**Authors:** Peter N Lee

**Affiliations:** 1 P N Lee Statistics and Computing Ltd, Sutton, Surrey SM2 5DA, United Kingdom

**Keywords:** Cancer, Oral disease, Circulatory disease, Tobacco, Smokeless

## Abstract

An earlier review summarized evidence relating use of snus (Swedish-type moist snuff) to health and to initiation and cessation of smoking. This update considers the effect recent publications on snus use and health have on the overall evidence. The additional evidence extends the list of neoplastic conditions unassociated with snus use (oropharynx, oesophagus, stomach, lung) to include colorectal cancer and acoustic neuroma, and further undermines the weakly-based argument that snus use increases the risk of pancreatic cancer, although there is a report of poorer cancer survival in users. It remains undemonstrated that “snuff-dipper’s lesion” increases risk of oral cancer, and recent publications add to the evidence that snus use has no effect on periodontitis or dental caries. Although onset of acute myocardial infarction is not adversely associated with snus use, there is some evidence of an association with reduced survival. Whether this is a direct effect of snus use or a result of confounding by socioeconomic status or other factors requires further investigation, as does a report of an increased risk of heart failure in snus users. Even if some adverse health effects of snus use do exist, it remains clear that they are far less than those of smoking.

## Review

### Background

Swedish-type moist snuff (“snus”) consists of finely ground air- or sun-cured tobacco, salt (sodium chloride), water, humidifying agents, chemical buffering agents (sodium carbonate), and food-grade flavourings. It is manufactured using a heat treatment process and, because of the manufacturing methods and selection of snus tobaccos, has stable levels of certain unwanted constituents, such as tobacco-specific nitrosamines, which are low compared to smokeless products prevalent on the US market, such as US-style moist snuff [1,2]. A pinch (or dip) of snus is placed between the gum and upper lip, often for 11 to 14 hours daily [3], in contrast to US finely cut moist snuff (or chewing tobacco) which is held (or chewed) in the gingival buccal area [4]. Use of snus involves nicotine exposure similar to and perhaps somewhat greater than that from smoking [5-11]. Although the sale of snus is banned in other EU countries, Sweden has a derogation, partly due to its long history of use.

In recent years there has been considerable interest in snus as a possible safer alternative to smoking. However, attention has been drawn to possible health effects of snus use. A review in 2008 for the EC [12] referred to its carcinogenicity, citing the pancreas as a major target organ, to the localized oral lesions it causes, and to possible risks of oral cancer and acute myocardial infarction (AMI). Following a series of reviews of the effects of smokeless tobacco on the various specific diseases of interest in which I was involved [4,13-16] I published, in 2011, a comprehensive review, including meta-analysis, of the epidemiological evidence relating snus to health [17]. My review concluded that “the evidence provides scant support for any major adverse health effects of snus”. Among my detailed conclusions I noted that the overall data showed no association of snus with heart disease or stroke or with various types of cancer (oropharynx, oesophagus, stomach, lung) and that the characteristic “snuff dipper’s lesion” [18] does not predict oral cancer. For pancreatic cancer, the evidence, as discussed more fully elsewhere [15] is limited and difficult to interpret, but overall does not show a statistically significant increased risk, either for never smokers, or for the whole population (with adjustment for smoking).

Since my comprehensive review [17] appeared I became aware of two more recent publications providing major new findings, one on pancreatic cancer [19], the other on AMI [20], diseases which are particularly relevant to the debate concerning the safety or otherwise of snus. It was therefore felt important to update my review, taking into account not only these two papers, but also other recent publications on snus use and health, in order to determine the effect they had on the overall conclusions. The update is limited to neoplastic conditions, circulatory disease (CID) and oral conditions.

## Methods

Methods are as previously described [17], with searches updated to 18th November 2012. Results are only summarized for health effects where new relevant publications could be identified.

## Results

Nine new relevant publications were identified [19-27].

### Pancreatic cancer

An earlier review of smokeless tobacco and risk of pancreatic cancer [15] considered nine North American and two Scandinavian studies. Seven of these provided estimates of the relative risk (RR) associated with smokeless tobacco use, based on data for smokers and nonsmokers combined, and with adjustment for smoking (the “smoking-adjusted relative risk”) and five provided estimates based on data for never smokers. Meta-analysis gave a combined estimate of the smoking-adjusted RR of 1.07 (95% confidence interval [CI] 0.71-1.60), and of 1.23 (95% CI 0.67-2.31) for the risk in never smokers. While there was little evidence of an increase in the North American studies, the two Scandinavian studies showed rather more indication of an increase. One [28] reported a significant increase (2.00, 95% CI 1.20-3.30) in never smokers, but not (0.90, 95% CI 0.70-1.20) in the smoking-adjusted analysis. The other [29], conducted in Norway, reported no increase (0.85, 95% CI 0.24-3.07) in never smokers, but did report a significant increase (1.67, 95% CI 1.12-1.50) in the smoking-adjusted analysis. This study has been criticized [30-33] for various reasons, including failure to adjust for alcohol, using an exposed group which included a completely different type of smokeless tobacco (skrá) as well as snus, and a very long follow-up with no updating of tobacco use. The EC report [12] misleadingly reported only the significant associations, leading to their conclusion that the pancreas was a main “target organ”, ignoring the fact that the combined evidence from those two studies did not show a significant increase, either for never smokers (1.61, 95% CI 0.77-3.34) or based on the smoking-adjusted RRs (1.20, 95% CI 0.66-2.20).

While there are no new studies on pancreatic cancer and snus, attention should be drawn to a recent paper [19] reporting the results of a pooled analysis relating pancreatic cancer to the use of forms of smokeless tobacco other than snus, based on 11 case-control studies, none in Scandinavia. Compared to never users of tobacco, the odds ratio (OR) was estimated as 0.98 (95% CI 0.75-1.27) for ever smokeless tobacco users and 0.62 (95% CI 0.37-1.04) for smokeless tobacco only users. Although it has been argued [34] that the case-control studies are subject to bias for various reasons, and that the two cohort studies [29,35] are “strong”, it would nevertheless seem that these results strengthen the argument that snus use is unassociated with an increased pancreatic cancer risk, especially since nitrosamine levels are substantially lower in snus than in other types of smokeless tobacco [36], nitrosamines being considered “the major and most abundant group of carcinogens” in smokeless tobacco products [12].

One must conclude that the evidence for an association of snus with pancreatic cancer, already weakly based before the pooled analysis [19], has become even more weakly based.

### Oral cancer

The direct epidemiological evidence previously considered [17] found no increased risk of oropharyngeal cancer associated with snus use, either in never smokers (RR 1.01, 95% CI 0.71-1.45, n = 4) or based on smoking-adjusted results (RR 0.97, 95% CI 0.68-1.37, n = 7), a result supported by long-term follow-up of 1115 individuals with snuff-dippers lesion, which observed no cancers at the site of lesions seen initially [37]. It is also consistent with evidence of a lack of increased risk associated with smokeless tobacco use in US populations in studies published since 1990 [4,16].

A recent paper [21] described 16 male patients with oral squamous cell carcinoma seen in seven Swedish hospitals, with the cancers reported to have occurred at the “exact anatomical location” where snus had been used for several years. The authors concluded that “Swedish snuff may not be a harmless alternative to smoking”. The difficulty in interpreting these results is that there is no control information on the distribution of sites among other oral cancer patients who used snus, or among patients who did not use snus, so that one cannot judge whether oral cancer is more likely than expected to be seen at the site of use. Even if snus does lead to some oral cancers at the site of use, the epidemiological evidence strongly suggests that any increased risk is small, and substantially less than that associated with smoking.

### Colorectal cancer

A recent study [22] is the first to report results relating to a possible association of snus with colon or rectal cancer. The study involved follow-up to 2007 of 336,381 male Swedish construction workers for whom detailed information on tobacco use at cohort entry had been collected in 1971-1992. Age- and BMI- adjusted RRs comparing pure snus users and non-users of any tobacco were presented by duration of use, but no evidence of an association was seen for cancers of the colon (based on n = 153 cases in users), or the rectum (n = 97), separate results being also presented for the right- and left-sided colon (n = 59 and 60 respectively). Anal cancers were also studied, but only one case was seen in users. Some limitations of the Construction Workers Study, which has reported results for many health endpoints, are discussed elsewhere [17].

### Acoustic neuroma

At the time of my earlier review [17], there was no evidence relating snus use to acoustic neuroma. A recent paper [24] described a study in Sweden conducted in 2002 to 2007, involving 451 cases and 710 population-based controls. Risk of acoustic neuroma, adjusted for education and tobacco use, was unassociated with current snus use (OR 0.94, 95% CI 0.57-1.55) or former snus use (OR 1.22, 95% CI 0.71-2.10). This contrasted with a marked reduction in risk associated with current smoking (OR 0.41, 95% CI 0.23-0.74), prompting the authors to suggest that protection may be conferred by a constituent of tobacco smoke other than nicotine.

### Survival from cancer

A recent paper [23] concerns survival from cancer among 40,320 male Swedish construction workers, 24,826 of whom had died. Compared to never tobacco users, an increased risk of death was evident in all tobacco groups studied after adjustment for age at diagnosis, period of diagnosis, cancer site and BMI, whether the death was from the primary cancer or another cause. The RR of overall mortality was 1.13 (95% CI 1.05-1.20) for pure snus users, 1.21 (1.17-1.25) for pure smokers and 1.17 (1.12-1.22) for combined users. For cause-specific mortality RRs for pure snus users (1.15, 1.05-1.26) and pure smokers (1.15, 1.10-1.21) were similar. The authors comment on their inability to control for stage of cancer at diagnosis, though did show that the increased mortality in pure snus users was similar in those with or without comorbidity. The authors noted that the mechanism behind the poorer survival in pure snus users warranted further investigation, and suggested that “nicotine may play a role”.

### Circulatory disease

Table four of my earlier review [17] summarized results relating snus use to ischaemic heart disease (IHD), AMI, stroke and any CID separately for never smokers and for the whole population with adjustment for smoking. None of the meta-analysis RR estimates were significant (p < 0.05) or exceeded 1.10 and I concluded that “although a small effect of snus on the incidence of CID cannot be excluded, this has not been demonstrated by the available epidemiological data”.

A recent paper [20] describes the results of a pooled analysis from eight prospective observational studies of the relationship of snus use to AMI, based on men who never smoked. Table 1 summarizes the total evidence relating snus use in male never smokers incorporating this latest data, and excluding those results reported earlier [38-40] which are superseded by the later individual study results given for the pooled analysis. Based on the 13 individual estimates of RR/OR the combined fixed effect estimate is 1.07 (95% CI 0.98-1.16). However, there is significant (p = 0.06) heterogeneity, and the random-effects estimate is 1.06 (95% CI 0.91-1.23). The major contributor to the heterogeneity is the individual estimate of 1.35 (95% CI 1.13-1.62) from the analysis of the Construction Workers Study based on interviews in 1971-1974. The heterogeneity may well have arisen because the results for this study relate to a period when the form of snus used was different. Also, during 1971-74, the data collected by questionnaire were limited for snus use and ambiguously coded for smoking [17]. Furthermore, it is the only estimate based only on fatal cases. Restricting attention to the other 12 estimates, the combined fixed effect estimate becomes 1.00 (95% CI 0.91-1.10) with no statistical evidence of heterogeneity.

**Table 1:** RR/OR of IHD/AMI for current (vs. never) snus use in never smoking Swedish men

**Source**	**Study**	**Type**^**a**^	**Period**^**b**^	**Cases**^**c**^	**End-point**^**d**^	**RR/OR (95% CI)**	**Adjustment factors**^**e**^
Bolinder et al. [41]	Construction Workers	PC	1971-74/1985	172	F	1.35 (1.13-1.62)^f^	age, res
Haglund et al. [42]	Survey of living conditions	PC	1988-89/2003	28	F + NF	0.77 (0.51-1.15)^g,h^	age, exe, hea, ill, res, ses
Wennberg et al. [43]	VIP^i^ and MONICA^j^	NCC	1985-99/1999	21	F + NF	0.82 (0.46-1.43)	age, bmi, cho, edu, lei, phy
Huhtasaari et al. [44]	1^st^ MONICA^j^ study	CC	1989-91	59	F + NF	0.89 (0.62-1.29)^g,h^	age
Huhtasaari et al. [45]	2^nd^ MONICA^j^ study	CC	1991-93	59	F + NF	0.93 (0.65-1.34)^f,h^	none
Hergens et al. [46]	Two counties	CC	1992-94	10	F + NF	0.73 (0.35-1.50)	age, res
Hansson et al. [20]	Construction Workers^k^	PC	1978-93/20y	309	F + NF	1.01 (0.90-1.14)	age
	Malmö diet and cancer^l^	PC	1991-96/13y	4	F + NF	1.00 (0.37-2.70)	age
	MONICA N.Sweden	PC	1986-2004/11y	7	F + NF	0.77 (0.35-1.69)	age
	National March Cohort	PC	1997/9y	0	F + NF	-	age
	SALT^m^	PC	1998-2002/8y	21	F + NF	1.56 (0.98-2.48)	age
	Stockholm Public Health	PC	2002/5y	5	F + NF	1.21 (0.48-3.08)	age
	Scania Public Health	PC	1991-2000/8y	8	F + NF	1.90 (0.90-4.00)	age
	WOLF^n^	PC	1992-98/9y	2	F + NF	3.30 (0.63-17.1)	age
Total	13 estimates				Fixed^o^	1.07 (0.98-1.16)	Het p = 0.06
					Random^p^	1.06 (0.91-1.23)	
Excluding [41]	12 estimates				Fixed^o^	1.00 (0.91-1.10)	Het p = 0.34
					Random^p^	1.00 (0.88-1.13)	

^a^PC = prospective cohort, NCC = nested case-control, CC = case-control.

^b^For case-control studies, the period of interviewing is shown. For prospective cohort or nested case-control studies, the baseline period is shown before the /, and either the final year of follow-up or, where this is not available, the mean person-years of follow-up after the /.

^c^The number of cases exposed to snus.

^d^F = fatal, NF = non-fatal.

^e^*Abbreviations used*: *bmi* body mass index, *cho* cholesterol level, *edu* education, *exe* exercise, *hea* self reported health, *ill* longstanding illness, *lei* leisure time, *phy* physical activity, *res* area of residence, *ses* socioeconomic status.

^f^Estimated from data in source article.

^g^Estimate is for current v non-current snus users.

^h^Estimate is for non-current smokers.

^i^VIP = Vasterbötten Intervention Program.

^j^ MONICA = Multinational Monitoring of trends and determinants in Cardiovascular disease.

^k^Results supersede those reported earlier [38].

^l^Results supersede those reported earlier [39].

^m^SALT = Screening across the lifespan twin study, results supersede those reported earlier [40].

^n^WOLF = Work, lipids and fibrinogen.

^o^“Fixed” – the result of the fixed effect meta-analysis is shown on the right followed by the between-study heterogeneity p value (“Het p”).

^p^“Random” – the result of the random-effects meta-analysis is shown on the right.

While these results seem consistent with a lack of effect of snus use on AMI, it should be noted that the pooled analysis [20], though reporting no association of snus use with the combined incidence of fatal and non-fatal AMI (RR 1.04, 95% CI 0.93-1.17) and no significant association with level or duration of use, did report that the short-term fatality rate “appeared increased” in snus users (OR 1.28, 95% CI 0.99-1.68). However the authors noted that this relationship “may be due to confounding by socioeconomic or life style factors”, and that confounding by level of education could not be addressed in this analysis due to lack of relevant data.

Results relating to the short-term fatality rate were not presented by study, but it seems clear that they largely depended on the findings from the Construction Workers Study which contributed 88% of the total person-years of follow-up. The results seem consistent with an earlier report from this study [38] which reported a significantly increased risk in never smokers associated with current snus use for fatal cases (RR 1.32, 95% CI 1.08-1.61) but a non-significantly decreased risk for non-fatal cases (RR 0.70, 95% CI 0.48-1.02). It should be noted that there are four other studies [42,43,45,46] which have separately reported results for fatal cases and either non-fatal cases or all cases combined. While (see Table 2) the estimates for fatal cases from those studies all have wide variability, it is interesting to note that in all these studies risks are also above 1.0 for fatal cases and below 1.0 for non-fatal cases. The combined RR/OR estimated from the five studies is 1.31 (95% CI 1.09-1.58) for fatal cases and 0.89 (95% CI 0.79-1.00) for non-fatal cases, with no evidence of heterogeneity. Taken at face-value, these results suggest that current snus use in never smokers may be associated with a slightly reduced risk of non-fatal AMI/IHD, but with an increased risk of fatal cases. Whether the increased risk of fatal cases reflects a direct effect of snus or an effect of confounding (e.g. a tendency for snus users to report disease later or have less medical care when they do) requires further investigation. In any case, it is evident that any true increase in risk of AMI resulting from snus use, if it exists, is very much less than that from smoking.

**Table 2:** **RR/OR of IHD/AMI for current (vs. never) snus use – by case fatality**^**a**^

**Source**	**Fatal**	**Non-fatal**^**b**^	**Ratio fatal to non-fatal**^**c**^
Haglund et al. ([42])^d^	1.15 (0.54-2.41)	0.65 (0.40-1.06)	1.77 (0.72-4.31)
Wennberg et al. ([43])^e^	1.12 (0.38-3.29)	0.73 (0.37-1.42)	1.54 (0.43-5.42)
Huhtasaari et al. ([45])^f^	1.50 (0.45-5.03)	0.36 (0.15-0.85)	4.22 (0.96-18.64)
Hergens et al. ([46])	1.70 (0.48-5.50)	0.59 (0.25-1.40)	2.88 (0.65-12.82)
Hergens et al. ([38])	1.32 (1.08-1.61)	0.94 (0.83-1.06)	1.40 (1.11-1.77)
Total^g^	1.31 (1.09-1.58)	0.89 (0.79-1.00)	1.48 (1.19-1.84)

^a^RR/ORs are based on data for male never smokers. Adjustment factors are as in Table 1 except where stated.

^b^For the first three studies, RR (CI) for non-fatal cases have been estimated from those for fatal cases and for combined fatal and non-fatal cases.

^c^RR (CI) for the ratio estimated from those for fatal and non-fatal cases.

^d^Estimates are for current v non-current snus use, based on non-current smokers.

^e^Fatal cases are fatal within 28 days of onset.

^f^Whereas the results in Table 1 for this study are unadjusted results for non-current smokers comparing current and never snus users, the results in Table 2 are results for non-current smokers comparing ever and never regular snus users with adjustment for hypertension, education, marital status, diabetes, cholesterol and family history of AMI.

^g^Fixed-effects estimates. There was no evidence of heterogeneity; with p = 0.98 for fatal, p = 0.10 for non-fatal and p = 0.55 for the ratio.

Another recent relevant publication [25] investigated the relationship between snus and risk of heart failure based on results from two Swedish cohorts. One was the Construction Workers Study, where an analysis in never smokers including 75 cases in current snus users reported a marginally significant RR of 1.28 (95% CI 1.00-1.64) after adjustment for age, BMI, region and AMI before baseline. However, no dose-response was seen with the daily amount of snus used. The other was the Uppsala Longitudinal Study. Here, the analysis included smokers and nonsmokers, and included 14 heart failure cases in snus users, the association again being marginally significant (RR 2.08, 95% CI 1.03-4.22). As the authors pointed out, the findings “need confirmation in further studies and underlying mechanisms remain to be elucidated”.

### Periodontitis and dental caries

The earlier review [17] briefly summarized evidence related to various aspects of non-neoplastic oral disease. Though there were some reports of increased risks of gingival disease [47] or of dental caries [48], more studies showed no effects on such diseases [49-52] and it was evident that any association of snus use with periodontal and gingival disease or with dental caries was not established.

These conclusions are supported by two recent publications [26,27] based on stratified random samples of the population of Jönköping, Sweden aged 20, 30, 40, 50, 60 and 70 years, taken in 1983, 1993 and 2003. After adjusting for age, sex and sociodemographic variables snus users were found to have no increased risk of periodontitis [26] or of dental caries [27].

## Discussion

Based on my previous review [17], I concluded that snus use is clearly much safer than smoking, and that any effects of snus use on the risk of cancer or CID, if they exist, are probably no more than 1% of that of smoking. I also noted that switching to using snus should improve the health prospects of those smokers unable or unwilling to relinquish nicotine, and that there is no good evidence that introducing snus into a population would encourage smoking initiation or discourage cessation.

While this update does not consider evidence relating snus use to initiation or cessation of smoking, it does provide further information on possible health effects associated with snus use. As regards neoplastic conditions, recent evidence extends the list of those unassociated with snus use to include colorectal cancer [22] and acoustic neuroma [24], and, for pancreatic cancer [19] adds further weight to undermine the claims [12,53] of an adverse effect of snus use, claims that have already been shown to be unsoundly based [15-17]. The recent publication [21], which reports some cases of oral cancers occurring at the site where snus has been placed, does not provide evidence of effect in the absence of control data on the distribution of sites of oral cancer among all snus users and among non-users. Here, the epidemiological evidence suggests that if there is an increased risk it is small, and much less than that associated with smoking. Despite the lack of evidence that snus use increases risk of cancer, the recent observation [23] that it is associated with poorer survival demands further investigation.

New publications on periodontitis [26] or dental caries [27] also add to the evidence that snus use has no effect on non-neoplastic oral lesions, other than causing “snuff-dippers lesion” [18], which has not been shown to be predictive of cancer.

Although the evidence remains clear that effects of snus use on risk of CID, if they exist, are much less than those of smoking, recent publications have provided evidence of increased risk in snus users of heart failure [25] and of case fatality from AMI [20]. More evidence on both these issues is needed. For AMI, this review presents evidence from five studies [38,42,43,45,46] that snus use, in never smokers, is associated with a somewhat reduced risk of non-fatal AMI, but with an increased risk of fatal AMI. The extent to which this difference reflects true effects of snus or uncontrolled confounding requires further investigation.

## Conclusions

Adverse health effects of snus use have not been clearly identified. The recent literature strengthens the evidence that any cancer risk (including that of pancreatic cancer) is at most minimal, and certainly much less than that associated with smoking. However, it does suggest that snus use might adversely affect survival from cancer. Although there is little evidence that snus use is associated with onset of AMI or stroke, reports of an increased risk of fatal AMI require further investigation.

## Abbreviations

AMI: Acute myocardial infarction; BMI: Body mass index; CI: Confidence interval; CID: Circulatory disease; IHD: Ischaemic heart disease; MONICA: Monitoring of cardiovascular disease; OR: Odds ratio; RR: Relative risk.

## Competing interests

The author is a long-term consultant to the tobacco industry. However, this is an independent scientific assessment, the views expressed being those of the author alone.

## Authors’ contributions

PNL was responsible for planning the study and writing the paper.
